# *N*,*N*,*N*′,*N*′-Tetra­methyl-*N*′′,*N*′′-dipropyl­guanidinium chloride–(2*Z*)-2,3-diamino­but-2-enedinitrile (1/1)

**DOI:** 10.1107/S1600536812023264

**Published:** 2012-05-31

**Authors:** Ioannis Tiritiris, Willi Kantlehner

**Affiliations:** a Institut für Organische Chemie, Universität Stuttgart, Pfaffenwaldring 55, 70569 Stuttgart, Germany; b Fakultät Chemie/Organische Chemie, Hochschule Aalen, Beethovenstrasse 1, D-73430 Aalen, Germany

## Abstract

In the crystal structure of the title compound, C_11_H_26_N_3_^+^·Cl^−^·C_4_H_4_N_4_, the (2*Z*)-2,3-diamino­but-2-ene-dinitrile (*Z*-DAMN) mol­ecules are connected with the chloride ions *via* N—H⋯Cl hydrogen bonds, forming ribbons running along the *a* axis. The guanidinium ions are located in between the ribbons formed by *Z*-DAMN mol­ecules and chloride ions.

## Related literature

For the crystal structure of (2*Z*)-2,3-diamino­but-2-enedinitrile, see: Penfold & Lipscomb (1961 ▶). For the synthesis of hexa­alkyl-substituted guanidinium chlorides, see: Kantlehner *et al.* (1984 ▶) and for the synthesis and crystal structures of hexa­alkyl-substituted guanidinium salts, see: Kantlehner *et al.* (2010 ▶). For studies on the water-absorption ability of guan­idin­ium salts, see: Kunkel (2008 ▶).
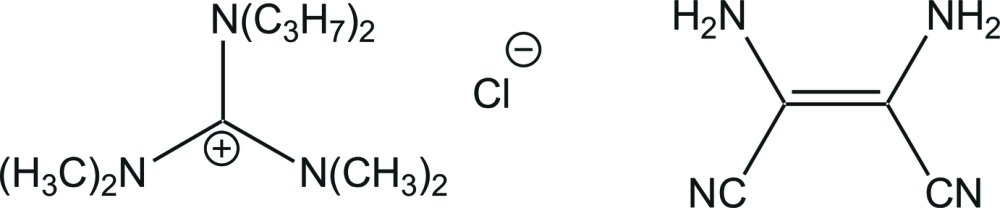


## Experimental

### 

#### Crystal data

C_11_H_26_N_3_^+^·Cl^−^·C_4_H_4_N_4_
*M* *_r_* = 343.91
Monoclinic, 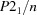

*a* = 8.5646 (3) Å
*b* = 24.6447 (9) Å
*c* = 9.5363 (4) Å
β = 101.341 (2)°
*V* = 1973.54 (13) Å^3^
*Z* = 4
Mo *K*α radiation
μ = 0.20 mm^−1^
*T* = 100 K
0.21 × 0.17 × 0.14 mm

#### Data collection

Bruker–Nonius KappaCCD diffractometer
8538 measured reflections
4901 independent reflections
3055 reflections with *I* > 2σ(*I*)
*R*_int_ = 0.052

#### Refinement

*R*[*F* ^2^ > 2σ(*F* ^2^)] = 0.047
*wR*(*F* ^2^) = 0.102
*S* = 1.01
4901 reflections
230 parameters
H atoms treated by a mixture of independent and constrained refinement
Δρ_max_ = 0.26 e Å^−3^
Δρ_min_ = −0.26 e Å^−3^

### 

Data collection: *COLLECT* (Hooft, 2004 ▶); cell refinement: *SCALEPACK* (Otwinowski & Minor, 1997 ▶); data reduction: *SCALEPACK*; program(s) used to solve structure: *SHELXS97* (Sheldrick, 2008 ▶); program(s) used to refine structure: *SHELXL97* (Sheldrick, 2008 ▶); molecular graphics: *DIAMOND* (Brandenburg & Putz, 2005 ▶); software used to prepare material for publication: *SHELXL97*.

## Supplementary Material

Crystal structure: contains datablock(s) I, global. DOI: 10.1107/S1600536812023264/kp2418sup1.cif

Structure factors: contains datablock(s) I. DOI: 10.1107/S1600536812023264/kp2418Isup2.hkl

Additional supplementary materials:  crystallographic information; 3D view; checkCIF report

## Figures and Tables

**Table 1:** Hydrogen-bond geometry (Å, °)

*D*—H⋯*A*	*D*—H	H⋯*A*	*D*⋯*A*	*D*—H⋯*A*
N4—H41⋯Cl1^i^	0.89 (2)	2.36 (2)	3.242 (2)	171 (2)
N4—H42⋯Cl1^ii^	0.87 (2)	2.48 (2)	3.351 (2)	174 (2)
N5—H51⋯Cl1	0.90 (2)	2.37 (2)	3.241 (2)	163 (2)
N5—H52⋯Cl1^ii^	0.86 (2)	2.48 (2)	3.333 (2)	173 (2)

Symmetry codes: (i) 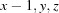; (ii) 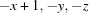.

**Fig. 1.**
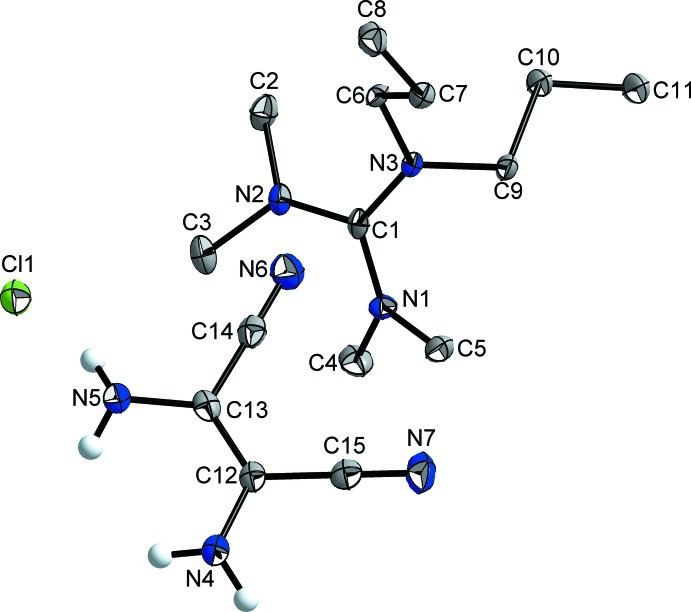
The structure of the title compound with atom labels and 50% probability displacement ellipsoids.

**Fig. 2.**
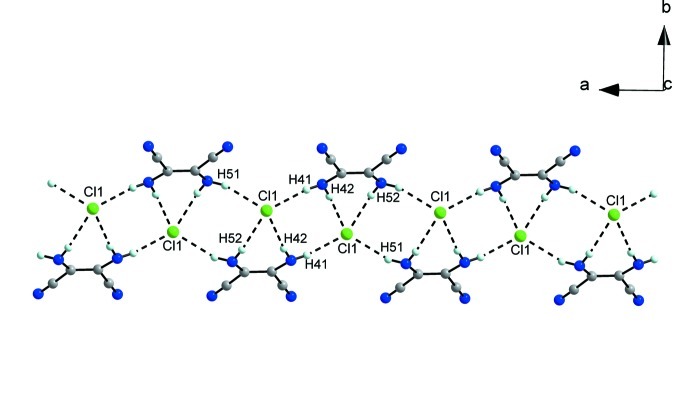
N–H···Cl hydrogen bonding system, ab-view. The hydrogen bonds are indicated by dashed lines.

**Fig. 3.**
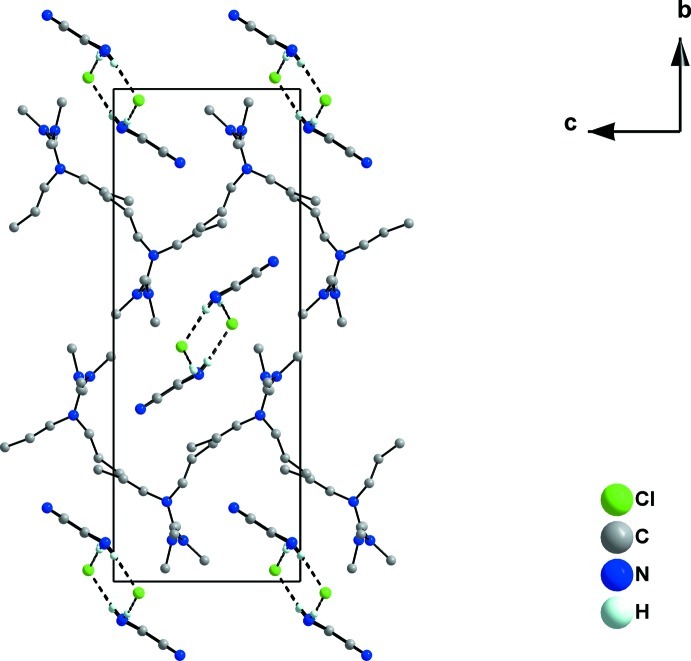
Packing diagram of the title compound, bc-view. The N–H···Cl hydrogen bonds are indicated by dashed lines.

## supplementary crystallographic information

### Comment

(2*Z*)-2,3-Diaminobut-2-enedinitrile (*Z*-DAMN), is considered to be the tetramer of hydrogen cyanide and its crystal structure has been determined more than fifty years ago (Penfold & Lipscomb, 1961). On the other hand the synthesis of hexaalkyl substituted guanidinium chlorides is well described in literature (Kantlehner *et al.*, 1984), but only scanty crystal structure data are available (Kantlehner *et al.*, 2010). For preparation of guanidinium chlorides, in first step *N*,*N*,*N'*,*N'*-tetraalkylureas are activated with phosgene to give chloroformamidinium chlorides, which in a next step react with secondary amines in the presence of triethylamine (Kantlehner *et al.*, 1984). A great disadvantage of the guanidinium chlorides is their hygroscopicity. The crystals obtained are liquefying very fast in air atmosphere and it has often proved difficult to determine their crystal structures. Recent studies showed that water absorption ability of guanidinium salts depends on the anion as well as on the cation. Salts with nucleophilic anions and short alkyl chains were found to be more water-soluble and hygroscopic (Kunkel, 2008). By recystallization of *N*,*N*,*N'*,*N'*-tetramethyl- *N''*,*N''*-dipropylguanidinium chloride from an acetonitrile solution containing equimolar amounts of *Z*-DAMN, 1:1 cocrystals have been obtained (Fig. 1). In contrast to the chloride salt, the title compound is no longer hygroscopic. The crystal structure analysis reveals that the *Z*-DAMN molecules are connected with the chloride ions *via* N–H···Cl hydrogen bonds, forming chains (Fig. 2) running along the *a* axis (Fig. 3). The Cl···H distances range between 2.36 (2) and 2.48 (2) Å, with N–H···Cl angles from 163 (2) to 174 (2)° (Tab. 1). The guanidinium ions are located inbetween the ribbons formed by *Z*-DAMN molecules and chloride ions (Fig. 3). They interact with the nitrogen atoms of both CN groups of *Z*-DAMN forming weak C–H···N hydrogen bonds [*d*(H···N) = 2.54 and 2.78 Å]. Prominent bond parameters in the guanidinium ion are: C1–N1 = 1.342 (2) Å, C1–N2 = 1.338 (2) Å and C1–N3 = 1.342 (2) Å. The N–C1–N angles are: 119.7 (2)° (N1–C1–N2), 119.9 (1)° (N2–C1–N3) and 120.4 (1)° (N1–C1–N3), which indicates a nearly ideal trigonal-planar surrounding of the carbon centre by the nitrogen atoms. The positive charge is completely delocalised on the CN_3_ plane. The geometrical parameters of the *Z*-DAMN molecule in the presented cocrystal, are very well comparable with the crystal structure data of the pure compound (Penfold & Lipscomb, 1961). The C–C double bond value is 1.359 (2) Å, the C–N single bonds are 1.386 (2) and 1.389 (2) Å, the C–C single bonds are 1.431 (2) and 1.441 (2) Å and both C–N triple bonds are 1.148 (2) Å.

### Experimental

The title compound was obtained by recrystallising *N*,*N*,*N'*,*N'*-tetramethyl- *N''*,*N''*-dipropylguanidinium-chloride from an acetonitrile solution containing equimolar amounts of (2*Z*)-2,3-diaminobut-2-enedinitrile. On slow evaporation of the solvent, the title compound crystallised in form of colourless, air stable single crystals.

### Refinement

The N-bound H atoms were located in a difference Fourier map and were refined freely [N—H = 0.86 (2)–0.90 (2) Å]. The hydrogen atoms of the methyl groups were allowed to rotate with a fixed angle around the C–N bond to best fit the experimental electron density, with *U*(H) set to 1.5 *U*_eq_(C) and d(C—H) = 0.98 Å. The remaining H atoms were placed in calculated positions with d(C—H) = 0.99 Å and were included in the refinement in the riding model approximation, with *U*(H) set to 1.2 *U*_eq_(C).

### Crystal data

**Table:**

C_11_H_26_N_3_^+^·Cl^−^·C_4_H_4_N_4_	*F*(000) = 744
*M**_r_* = 343.91	*D*_x_ = 1.158 Mg m^−^^3^
Monoclinic, *P*2_1_/*n*	Mo *K*α radiation, λ = 0.71073 Å
Hall symbol: -P 2yn	Cell parameters from 4700 reflections
*a* = 8.5646 (3) Å	θ = 0.4–28.3°
*b* = 24.6447 (9) Å	µ = 0.20 mm^−^^1^
*c* = 9.5363 (4) Å	*T* = 100 K
β = 101.341 (2)°	Lath-shaped, colourless
*V* = 1973.54 (13) Å^3^	0.21 × 0.17 × 0.14 mm
*Z* = 4	

### Data collection

**Table:**

Bruker–Nonius KappaCCD diffractometer	3055 reflections with *I* > 2σ(*I*)
Radiation source: sealed tube	*R*_int_ = 0.052
Graphite monochromator	θ_max_ = 28.3°, θ_min_ = 1.7°
φ scans, and ω scans	*h* = −11→11
8538 measured reflections	*k* = −32→30
4901 independent reflections	*l* = −12→12

### Refinement

**Table:**

Refinement on *F*^2^	Primary atom site location: structure-invariant direct methods
Least-squares matrix: full	Secondary atom site location: difference Fourier map
*R*[*F*^2^ > 2σ(*F*^2^)] = 0.047	Hydrogen site location: difference Fourier map
*wR*(*F*^2^) = 0.102	H atoms treated by a mixture of independent and constrained refinement
*S* = 1.01	*w* = 1/[σ^2^(*F*_o_^2^) + (0.043*P*)^2^] where *P* = (*F*_o_^2^ + 2*F*_c_^2^)/3
4901 reflections	(Δ/σ)_max_ < 0.001
230 parameters	Δρ_max_ = 0.26 e Å^−^^3^
0 restraints	Δρ_min_ = −0.26 e Å^−^^3^

### Special details

**Table:**

Geometry. All e.s.d.'s (except the e.s.d. in the dihedral angle between two l.s. planes) are estimated using the full covariance matrix. The cell e.s.d.'s are taken into account individually in the estimation of e.s.d.'s in distances, angles and torsion angles; correlations between e.s.d.'s in cell parameters are only used when they are defined by crystal symmetry. An approximate (isotropic) treatment of cell e.s.d.'s is used for estimating e.s.d.'s involving l.s. planes.
Refinement. Refinement of *F*^2^ against ALL reflections. The weighted *R*-factor *wR* and goodness of fit *S* are based on *F*^2^, conventional *R*-factors *R* are based on *F*, with *F* set to zero for negative *F*^2^. The threshold expression of *F*^2^ > σ(*F*^2^) is used only for calculating *R*-factors(gt) *etc*. and is not relevant to the choice of reflections for refinement. *R*-factors based on *F*^2^ are statistically about twice as large as those based on *F*, and *R*- factors based on ALL data will be even larger.

### Fractional atomic coordinates and isotropic or equivalent isotropic displacement parameters (Å^2^)

**Table:**

	*x*	*y*	*z*	*U*_iso_*/*U*_eq_	
Cl1	0.77349 (4)	0.023155 (18)	0.13502 (5)	0.02555 (13)	
C1	0.73690 (17)	0.11012 (7)	0.67572 (16)	0.0157 (4)	
N1	0.60198 (14)	0.08427 (6)	0.68672 (14)	0.0179 (3)	
N2	0.85119 (14)	0.08348 (6)	0.62646 (14)	0.0180 (3)	
N3	0.75830 (14)	0.16234 (5)	0.71465 (13)	0.0145 (3)	
C2	1.02067 (17)	0.09594 (8)	0.67499 (18)	0.0241 (4)	
H2A	1.0601	0.1154	0.5994	0.036*	
H2B	1.0806	0.0621	0.6971	0.036*	
H2C	1.0346	0.1186	0.7610	0.036*	
C3	0.8157 (2)	0.04293 (8)	0.51327 (19)	0.0274 (4)	
H3A	0.8484	0.0070	0.5526	0.041*	
H3B	0.8738	0.0517	0.4373	0.041*	
H3C	0.7010	0.0428	0.4739	0.041*	
C4	0.6013 (2)	0.02679 (7)	0.72316 (19)	0.0270 (4)	
H4A	0.5649	0.0055	0.6361	0.041*	
H4B	0.5293	0.0208	0.7898	0.041*	
H4C	0.7093	0.0155	0.7681	0.041*	
C5	0.44834 (17)	0.11190 (7)	0.66526 (18)	0.0228 (4)	
H5A	0.4136	0.1149	0.7569	0.034*	
H5B	0.3695	0.0911	0.5979	0.034*	
H5C	0.4587	0.1483	0.6265	0.034*	
C6	0.83711 (16)	0.19998 (7)	0.63049 (16)	0.0158 (4)	
H6A	0.8609	0.1807	0.5461	0.019*	
H6B	0.9394	0.2122	0.6895	0.019*	
C7	0.73418 (17)	0.24919 (7)	0.58080 (17)	0.0180 (4)	
H7A	0.6240	0.2372	0.5412	0.022*	
H7B	0.7311	0.2731	0.6636	0.022*	
C8	0.7983 (2)	0.28072 (8)	0.46745 (18)	0.0260 (4)	
H8A	0.9067	0.2932	0.5071	0.039*	
H8B	0.7297	0.3121	0.4373	0.039*	
H8C	0.7999	0.2572	0.3849	0.039*	
C9	0.70155 (17)	0.18362 (7)	0.84042 (16)	0.0174 (4)	
H9A	0.6533	0.1537	0.8867	0.021*	
H9B	0.6182	0.2113	0.8089	0.021*	
C10	0.83651 (17)	0.20876 (8)	0.94804 (17)	0.0217 (4)	
H10A	0.8801	0.2402	0.9038	0.026*	
H10B	0.9230	0.1818	0.9748	0.026*	
C11	0.7796 (2)	0.22732 (8)	1.08166 (18)	0.0302 (5)	
H11A	0.6972	0.2552	1.0558	0.045*	
H11B	0.8694	0.2424	1.1502	0.045*	
H11C	0.7353	0.1963	1.1250	0.045*	
C12	0.20196 (18)	0.09908 (7)	0.15547 (17)	0.0185 (4)	
C13	0.36014 (18)	0.10073 (7)	0.15344 (17)	0.0186 (4)	
N4	0.08854 (18)	0.07578 (7)	0.04879 (17)	0.0255 (4)	
H41	−0.001 (2)	0.0652 (9)	0.075 (2)	0.048 (6)*	
H42	0.129 (2)	0.0521 (9)	−0.002 (2)	0.033 (6)*	
N5	0.42670 (19)	0.07966 (7)	0.04344 (16)	0.0216 (3)	
H51	0.530 (2)	0.0716 (8)	0.0693 (19)	0.034 (5)*	
H52	0.369 (2)	0.0554 (9)	−0.006 (2)	0.031 (6)*	
C14	0.4658 (2)	0.12854 (7)	0.26529 (19)	0.0228 (4)	
N6	0.55281 (18)	0.15012 (7)	0.35497 (17)	0.0334 (4)	
C15	0.14409 (19)	0.12679 (7)	0.26810 (19)	0.0227 (4)	
N7	0.09678 (18)	0.14926 (7)	0.35655 (17)	0.0344 (4)	

### Atomic displacement parameters (Å^2^)

**Table:**

	*U*^11^	*U*^22^	*U*^33^	*U*^12^	*U*^13^	*U*^23^
Cl1	0.0218 (2)	0.0282 (3)	0.0271 (3)	0.00105 (18)	0.00570 (16)	−0.0083 (2)
C1	0.0188 (8)	0.0192 (10)	0.0093 (8)	0.0032 (7)	0.0031 (6)	0.0015 (7)
N1	0.0192 (7)	0.0153 (8)	0.0206 (8)	−0.0030 (6)	0.0070 (5)	0.0012 (6)
N2	0.0191 (7)	0.0201 (9)	0.0155 (7)	0.0051 (6)	0.0049 (5)	−0.0011 (6)
N3	0.0152 (6)	0.0164 (8)	0.0133 (7)	−0.0007 (6)	0.0059 (5)	−0.0011 (6)
C2	0.0192 (8)	0.0309 (12)	0.0221 (10)	0.0089 (7)	0.0035 (7)	0.0012 (8)
C3	0.0345 (9)	0.0246 (11)	0.0240 (10)	0.0059 (8)	0.0075 (8)	−0.0061 (8)
C4	0.0372 (10)	0.0196 (11)	0.0256 (10)	−0.0056 (8)	0.0095 (8)	0.0016 (8)
C5	0.0164 (8)	0.0292 (12)	0.0236 (10)	−0.0022 (7)	0.0057 (7)	−0.0022 (8)
C6	0.0129 (7)	0.0198 (10)	0.0161 (9)	−0.0028 (7)	0.0064 (6)	0.0001 (7)
C7	0.0183 (8)	0.0185 (10)	0.0179 (9)	−0.0007 (7)	0.0053 (6)	−0.0006 (7)
C8	0.0348 (9)	0.0234 (11)	0.0212 (10)	−0.0016 (8)	0.0091 (7)	−0.0007 (8)
C9	0.0158 (8)	0.0236 (10)	0.0144 (9)	−0.0004 (7)	0.0066 (6)	−0.0019 (7)
C10	0.0197 (8)	0.0283 (11)	0.0170 (9)	−0.0029 (7)	0.0039 (6)	−0.0039 (8)
C11	0.0277 (9)	0.0442 (13)	0.0200 (10)	−0.0104 (8)	0.0075 (7)	−0.0121 (9)
C12	0.0262 (9)	0.0147 (10)	0.0160 (9)	0.0010 (7)	0.0075 (7)	0.0030 (7)
C13	0.0272 (9)	0.0121 (9)	0.0168 (9)	0.0022 (7)	0.0047 (7)	0.0030 (7)
N4	0.0226 (8)	0.0295 (11)	0.0259 (9)	−0.0003 (7)	0.0087 (7)	−0.0065 (8)
N5	0.0232 (8)	0.0219 (10)	0.0203 (8)	−0.0004 (7)	0.0053 (6)	−0.0022 (7)
C14	0.0279 (9)	0.0210 (10)	0.0215 (10)	0.0010 (8)	0.0097 (8)	0.0034 (8)
N6	0.0371 (9)	0.0377 (11)	0.0258 (9)	−0.0062 (8)	0.0071 (7)	−0.0044 (8)
C15	0.0280 (9)	0.0196 (11)	0.0217 (10)	−0.0014 (8)	0.0078 (7)	0.0052 (8)
N7	0.0437 (9)	0.0377 (11)	0.0257 (9)	0.0010 (8)	0.0165 (7)	−0.0023 (8)

### Geometric parameters (Å, º)

**Table:**

C1—N2	1.3381 (19)	C7—H7A	0.9900
C1—N1	1.3415 (19)	C7—H7B	0.9900
C1—N3	1.342 (2)	C8—H8A	0.9800
N1—C4	1.459 (2)	C8—H8B	0.9800
N1—C5	1.4598 (19)	C8—H8C	0.9800
N2—C3	1.459 (2)	C9—C10	1.519 (2)
N2—C2	1.4667 (19)	C9—H9A	0.9900
N3—C6	1.4742 (19)	C9—H9B	0.9900
N3—C9	1.4761 (19)	C10—C11	1.522 (2)
C2—H2A	0.9800	C10—H10A	0.9900
C2—H2B	0.9800	C10—H10B	0.9900
C2—H2C	0.9800	C11—H11A	0.9800
C3—H3A	0.9800	C11—H11B	0.9800
C3—H3B	0.9800	C11—H11C	0.9800
C3—H3C	0.9800	C12—C13	1.359 (2)
C4—H4A	0.9800	C12—N4	1.386 (2)
C4—H4B	0.9800	C12—C15	1.441 (2)
C4—H4C	0.9800	C13—N5	1.389 (2)
C5—H5A	0.9800	C13—C14	1.431 (2)
C5—H5B	0.9800	N4—H41	0.89 (2)
C5—H5C	0.9800	N4—H42	0.87 (2)
C6—C7	1.519 (2)	N5—H51	0.90 (2)
C6—H6A	0.9900	N5—H52	0.86 (2)
C6—H6B	0.9900	C14—N6	1.148 (2)
C7—C8	1.519 (2)	C15—N7	1.148 (2)
			
N2—C1—N1	119.70 (15)	C8—C7—H7A	109.4
N2—C1—N3	119.87 (14)	C6—C7—H7A	109.4
N1—C1—N3	120.44 (13)	C8—C7—H7B	109.4
C1—N1—C4	121.56 (13)	C6—C7—H7B	109.4
C1—N1—C5	122.28 (14)	H7A—C7—H7B	108.0
C4—N1—C5	116.15 (13)	C7—C8—H8A	109.5
C1—N2—C3	122.37 (13)	C7—C8—H8B	109.5
C1—N2—C2	122.24 (14)	H8A—C8—H8B	109.5
C3—N2—C2	115.24 (13)	C7—C8—H8C	109.5
C1—N3—C6	120.34 (13)	H8A—C8—H8C	109.5
C1—N3—C9	121.10 (13)	H8B—C8—H8C	109.5
C6—N3—C9	118.56 (13)	N3—C9—C10	111.44 (11)
N2—C2—H2A	109.5	N3—C9—H9A	109.3
N2—C2—H2B	109.5	C10—C9—H9A	109.3
H2A—C2—H2B	109.5	N3—C9—H9B	109.3
N2—C2—H2C	109.5	C10—C9—H9B	109.3
H2A—C2—H2C	109.5	H9A—C9—H9B	108.0
H2B—C2—H2C	109.5	C9—C10—C11	111.21 (12)
N2—C3—H3A	109.5	C9—C10—H10A	109.4
N2—C3—H3B	109.5	C11—C10—H10A	109.4
H3A—C3—H3B	109.5	C9—C10—H10B	109.4
N2—C3—H3C	109.5	C11—C10—H10B	109.4
H3A—C3—H3C	109.5	H10A—C10—H10B	108.0
H3B—C3—H3C	109.5	C10—C11—H11A	109.5
N1—C4—H4A	109.5	C10—C11—H11B	109.5
N1—C4—H4B	109.5	H11A—C11—H11B	109.5
H4A—C4—H4B	109.5	C10—C11—H11C	109.5
N1—C4—H4C	109.5	H11A—C11—H11C	109.5
H4A—C4—H4C	109.5	H11B—C11—H11C	109.5
H4B—C4—H4C	109.5	C13—C12—N4	123.97 (15)
N1—C5—H5A	109.5	C13—C12—C15	119.10 (15)
N1—C5—H5B	109.5	N4—C12—C15	116.72 (14)
H5A—C5—H5B	109.5	C12—C13—N5	123.89 (15)
N1—C5—H5C	109.5	C12—C13—C14	119.35 (15)
H5A—C5—H5C	109.5	N5—C13—C14	116.64 (14)
H5B—C5—H5C	109.5	C12—N4—H41	115.9 (14)
N3—C6—C7	111.86 (11)	C12—N4—H42	112.8 (12)
N3—C6—H6A	109.2	H41—N4—H42	114.6 (19)
C7—C6—H6A	109.2	C13—N5—H51	114.0 (12)
N3—C6—H6B	109.2	C13—N5—H52	113.2 (12)
C7—C6—H6B	109.2	H51—N5—H52	115.6 (17)
H6A—C6—H6B	107.9	N6—C14—C13	178.7 (2)
C8—C7—C6	111.19 (13)	N7—C15—C12	179.1 (2)
			
N2—C1—N1—C4	33.9 (2)	N1—C1—N3—C9	37.9 (2)
N3—C1—N1—C4	−145.70 (15)	C1—N3—C6—C7	124.34 (15)
N2—C1—N1—C5	−147.08 (15)	C9—N3—C6—C7	−54.64 (17)
N3—C1—N1—C5	33.4 (2)	N3—C6—C7—C8	−166.97 (13)
N1—C1—N2—C3	36.4 (2)	C1—N3—C9—C10	122.97 (15)
N3—C1—N2—C3	−143.99 (16)	C6—N3—C9—C10	−58.06 (18)
N1—C1—N2—C2	−148.24 (15)	N3—C9—C10—C11	−176.32 (15)
N3—C1—N2—C2	31.3 (2)	N4—C12—C13—N5	0.6 (3)
N2—C1—N3—C6	39.34 (19)	C15—C12—C13—N5	−174.05 (16)
N1—C1—N3—C6	−141.10 (14)	N4—C12—C13—C14	176.48 (16)
N2—C1—N3—C9	−141.71 (14)	C15—C12—C13—C14	1.8 (2)

### Hydrogen-bond geometry (Å, º)

**Table:**

*D*—H···*A*	*D*—H	H···*A*	*D*···*A*	*D*—H···*A*
N4—H41···Cl1^i^	0.89 (2)	2.36 (2)	3.242 (2)	171 (2)
N4—H42···Cl1^ii^	0.87 (2)	2.48 (2)	3.351 (2)	174 (2)
N5—H51···Cl1	0.90 (2)	2.37 (2)	3.241 (2)	163 (2)
N5—H52···Cl1^ii^	0.86 (2)	2.48 (2)	3.333 (2)	173 (2)

Symmetry codes: (i) *x*−1, *y*, *z*; (ii) −*x*+1, −*y*, −*z*.
